# An NQO1-Initiated and p53-Independent Apoptotic Pathway Determines the Anti-Tumor Effect of Tanshinone IIA against Non-Small Cell Lung Cancer

**DOI:** 10.1371/journal.pone.0042138

**Published:** 2012-07-27

**Authors:** Fang Liu, Guo Yu, Guangji Wang, Huiying Liu, Xiaolan Wu, Qiong Wang, Miao Liu, Ke Liao, Mengqiu Wu, Xuefang Cheng, Haiping Hao

**Affiliations:** State Key Laboratory of Natural Medicines, Key Lab of Drug Metabolism and Pharmacokinetics, China Pharmaceutical University, Nanjing, China; University of Windsor, Canada

## Abstract

NQO1 is an emerging and promising therapeutic target in cancer therapy. This study was to determine whether the anti-tumor effect of tanshinone IIA (TSA) is NQO1 dependent and to elucidate the underlying apoptotic cell death pathways. NQO1^+^ A549 cells and isogenically matched NQO1 transfected and negative H596 cells were used to test the properties and mechanisms of TSA induced cell death. The in vivo anti-tumor efficacy and the tissue distribution properties of TSA were tested in tumor xenografted nude mice. We observed that TSA induced an excessive generation of ROS, DNA damage, and dramatic apoptotic cell death in NQO1^+^ A549 cells and H596-NQO1 cells, but not in NQO1^−^ H596 cells. Inhibition or silence of NQO1 as well as the antioxidant NAC markedly reversed TSA induced apoptotic effects. TSA treatment significantly retarded the tumor growth of A549 tumor xenografts, which was significantly antagonized by dicoumarol co-treatment in spite of the increased and prolonged TSA accumulations in tumor tissues. TSA activated a ROS triggered, p53 independent and caspase dependent mitochondria apoptotic cell death pathway that is characterized with increased ratio of Bax to Bcl-xl, mitochondrial membrane potential disruption, cytochrome c release, and subsequent caspase activation and PARP-1 cleavage. The results of these findings suggest that TSA is a highly specific NQO1 target agent and is promising in developing as an effective drug in the therapy of NQO1 positive NSCLC.

## Introduction

Non-small cell lung cancer (NSCLC) accounts for approximately 80∼85% of all cases of lung cancer, and is the most common cause of death in men and second to breast cancer in women [1]. Combination chemotherapy, usually platinum-based, is currently the first-line therapy of choice for NSCLCs. However, the prognosis for patients with advanced NSCLC remains poor with a median survival time of 8 to11 months and a 1-year survival rate of 30% [2], [3]. The long term survival (5-year) rate was even poor at around 15% [4]. The recent development of various molecular target drugs and their combination with chemotherapy drugs improves the outcome of NSCLC therapy; however, it remains disappointing in the therapy of advanced NSCLC. Obviously, there is an urgent need to identify new therapeutic targets and to develop tumor-selective chemotherapeutic drugs specific for NSCLCs.

NAD(P)H:quinone oxidoreductase (NQO1, EC 1.6.99.2) is a cytosolic flavoenzyme that catalyzes the obligatory two-electron reduction of a variety of quinone substrates, using both NADH and NADPH as electron donors [5]. Originally, NQO1 was widely believed to be a detoxification enzyme in view of its two-electron reduction property, bypassing the one-electron reduction producing unstable and highly reactive semiquinone [6], [7]. Lately, it was found that some quinones such as mitomycin C, streptonigrin, E09, and RH1 [8], [9], [10], [11], [12] were bioactivated by NQO1. The bioactivation property of NQO1 promises it an ideal target for developing anti-tumor drugs, because various human tumors [13] have elevated NQO1 activities. In the case of lung tumors, NQO1 activity is increased up to 80-fold in NSCLC tumors relative to normal lung, and 20∼35-fold relative to SCLC cell lines [14]. Such a differentiated expression mode of NQO1 between tumors and normal tissues suggests that NQO1 target drugs would be highly selective in killing tumor cells while saving normal tissues. RH1 is a drug candidate bioactivated by NQO1 to produce hydroquinone in the activation of the aziridine rings and subsequent DNA alkylation and interstrand cross-linking. In this case, NQO1 is utilized as a tumor selective enzyme to bioactivate the prodrug and thus to realize a tumor specific toxicity. In addition to its property as an oxidoreductase, NQO1 has been also found directly involved in stabilizing the vital tumor suppressors p53/p73/p33 [15], [16]. Moreover, NQO1 polymorphism that leads to the enzyme inactivity has been found to be a strong prognostic and predictive factor in the poor outcome of breast cancer [17]. These findings suggest that the pharmacological role of NQO1 is far beyond its enzymatic activity on reducing quinones. Taking together, it would be of high interest to determine the therapeutic potentials and underlying mechanisms of NQO1 target agents on tumors. β-Lapachone (Lap), a well studied NQO1 substrate, has been identified as a promising agent for various cancer therapy [18]. However, repeated oral treatment of Lap induces anemia in both rats and humans which may greatly limit its application [19], [20]. Another limitation of Lap is its NQO1 specific activity can only be realized within a relatively narrow concentration range and exposure time window [18]; it is questionable whether such a condition can be well controlled in the in vivo states.

Tanshinone IIA (TSA) was a derivative of phenanthrene-quinone isolated from Salvia miltiorrhiza (Danshen, in Chinese), a widely used herbal medicine. Although its traditional use mainly focused in cardiovascular and cerebrovascular diseases [21], [22], [23], [24], the anti-tumor activity of TSA and other structurally similar tanshinones were confirmed during the past decades [25], [26], [27]. Various studies showed that TSA exerted cytotoxic effects against a wide range of human tumor cell lines [28]. In spite of previous efforts on disclosing its mechanisms [29], [30], the molecular target of TSA to elicit its anti-tumor effect remains elusive. We have recently found that NQO1 catalyzed quinone reduction followed by immediate glucuronidation is the predominant metabolic pathway of TSA in both rats [31] and humans [32]. NQO1 reduces TSA to form a highly unstable catechol intermediate which auto-oxidizes back to parent TSA and constitutes a futile redox cycle producing oxidative stress [31]. These findings prompted us to hypothesize that NQO1 may be the primary intracellular target of TSA in cancer cells.

The present study aims to determine whether NQO1 plays a pivotal role on initiating the anti-tumor effect of TSA both in vitro and in vivo. Isogenically matched NQO1^+^ and NQO1^−^ human H596 and NQO1 high expression human A549 NSCLC cell lines were applied to determine the NQO1 dependent cytotoxicity, apoptotic effect, ROS production, DNA damage, and cellular uptake of TSA. We demonstrate that TSA is a promising NQO1 specific target agent, which induces apoptotic cell death of NSCLC cells via a unique NQO1-initiated and ROS-mediated activation of a p53-independent but caspase-dependent mitochondrial apoptotic pathway.

## Results

### TSA induced cytotoxicity is NQO1 dependent and p53-independent

**Figure 1:**
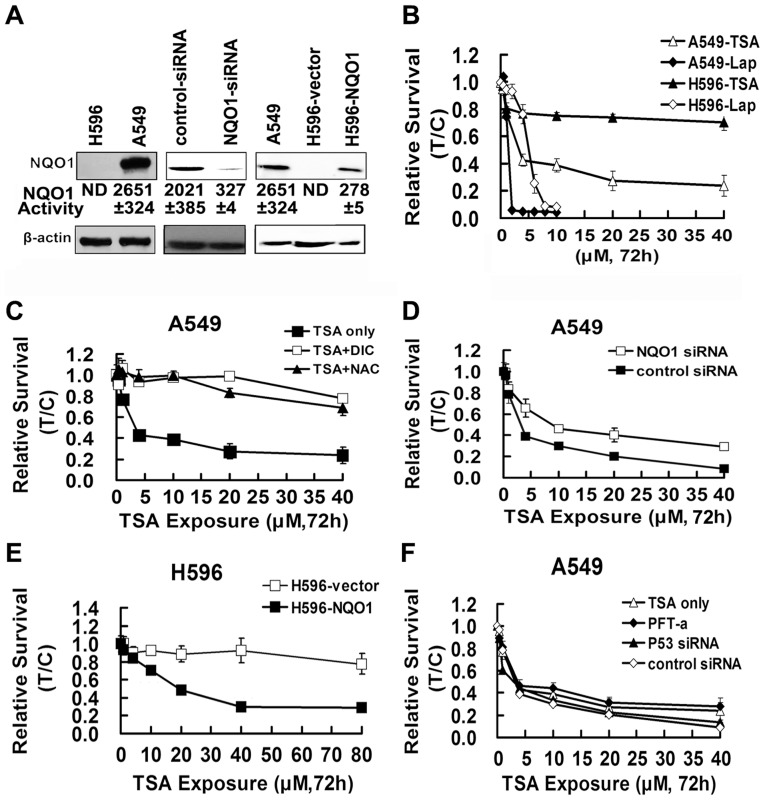
TSA induced cytotoxicity is NQO1 dependent, ROS mediated, and p53-independent (MTT assay). A, protein levels and enzyme activities of NQO1 in NSCLC cells. NQO1 activity <1.0 nmol⋅min^−1^⋅µg^−1^ was indicated non-detectable (ND). B, cytotoxicity of TSA and Lap in NSCLC cells; cells were exposed to gradient concentrations of TSA (0.4–40 µM) or Lap (0.5–10 µM) for 72 h. C, effects of NAC or DIC pretreatment; A549 cells were pretreated with 5 mM of NAC for 1 h or 10 µM of DIC for 30 min. D, effects of NQO1 silence in A549 cells. E, TSA cytotoxicity in H596 and NQO1-transfected H596 cells. F, effect of P53 inhibition or silence; A549 cells were pretreated with 10 µM of PFT-α for 5 h or pretreated with P53 siRNA. Data are shown as mean ± SE of three independent experiments.

To determine the role of NQO1, TSA cytotoxicity assays were performed in NQO1 high expression A549 cells, NQO1 negative and transfected H596 cells; NQO1 protein levels and enzyme activities in NSCLC cells are shown in Fig. 1A. Protein expression and enzyme activity of NQO1 could not be detected in H596 cells; the NQO1 enzyme activity in H596-NQO1 cells is much lower than that in A549 cells, which is consistent with previous findings [18]. TSA induced a concentration dependent cytotoxicity in A549 cells with an IC50 value at 5.32 µM. In contrast, NQO1 negative H596 cells were largely resistant to TSA cytotoxicity with an IC50>40 µM (Fig. 1B). NQO1 inhibition by the specific NQO1 inhibitor dicoumarol (DIC,10 µM) could largely prevent TSA's cytotoxicity in A549 cells; NQO1 siRNA knockdown could also abrogate the cytotoxic effect of TSA, albeit to a lesser extent to that by DIC (Fig. 1C, D). The transfection of NQO1 to H596 cells restored its susceptibility to TSA induced cytotoxicity. The IC50 of TSA in H596-NQO1 and in H596-vector cell lines is of 23.56 µM and >80 µM, respectively (Fig. 1E). Of note, the IC50 values in A549 cells were much lower than that in H596-NQO1 cells, which is in agreement with the difference of enzyme activities between these two cell lines. All these results strongly suggest that TSA induced cytotoxicity is NQO1 dependent. In addition, it seems that TSA showed better NQO1 target specificity than Lap; the IC50 value difference between A549 cells and H596 cells for TSA (5.32 µM VS>40 µM) is much higher than that for Lap (1.94 µM VS 3.93 µM). Moreover, the pretreatment with typical p53 inhibitor pifithrin-α (PFT-α) [33] and the p53 silence by siRNA had negligible effect on TSA induced cytotoxicity (Fig. 1F), suggesting TSA induced cytotoxicity in a p53-independent manner.

### TSA induces NQO1 dependent and p53-independent apoptotic cell death

**Figure 2:**
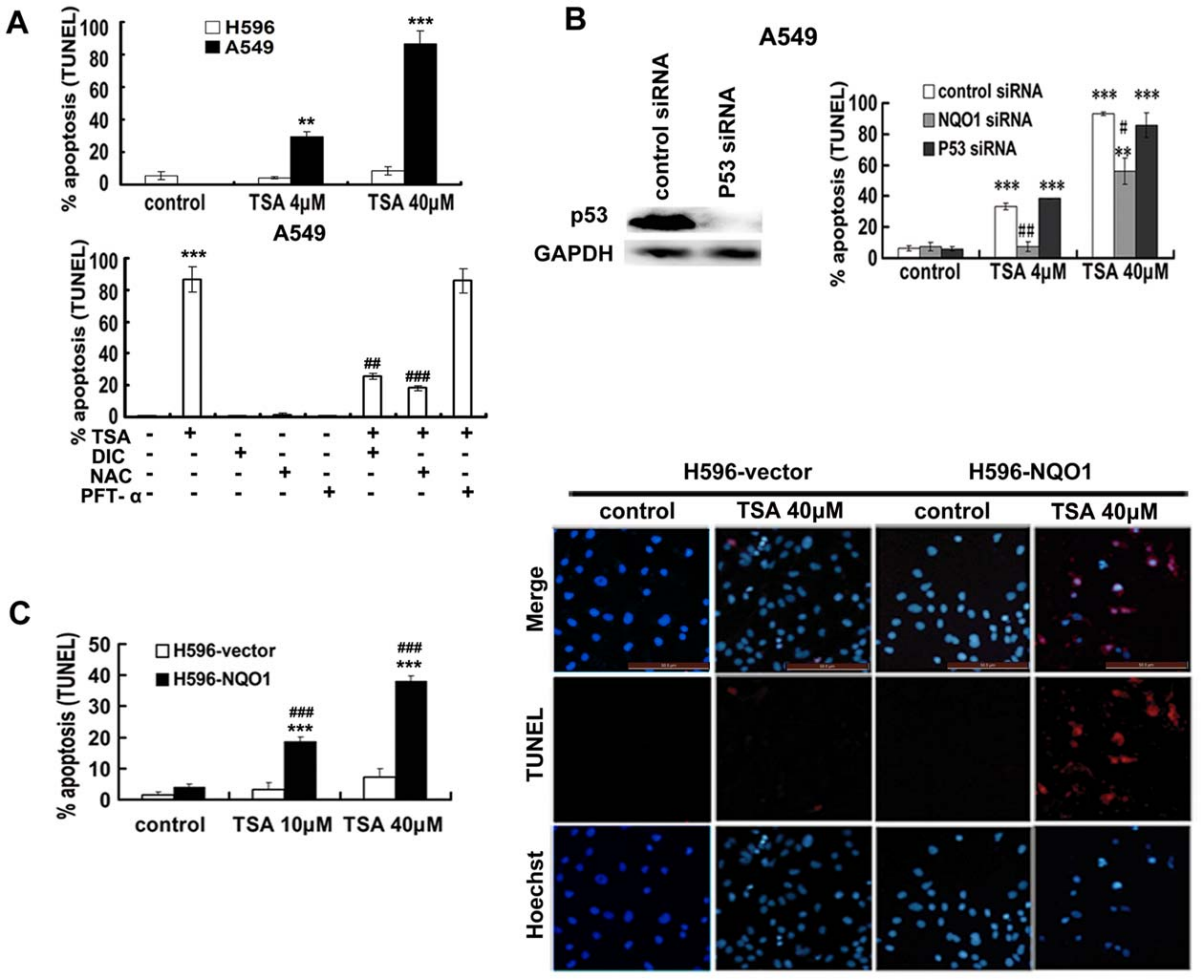
TSA induced apoptosis is NQO1-dependent, ROS-mediated, and p53-independent (TUNEL assay). A, upper, A549 cells and H596 cells were exposed to indicated concentration of TSA for 48 h; lower, A549 cells were pretreated with 10 µM of DIC for 30 min, 5 mM of NAC for 1 h, or 10 µM of PFT-α for 5 h and then exposed to 40 µM of TSA for 48 h. B, effects of NQO1 or p53 silence on TSA induced apoptosis in A549 cells. C, H596-vector and H596-NQO1 cells were exposed to TSA for 48 h; the TUNEL assay was performed using Click-iT TUNEL Alexa Fluor 594 Imaging Assay Kit. Eight to ten fields were examined in each experiment, the TUNEL signal is shown in red and Hoechst 33342 nuclear stain is shown in blue. Right are the representative images of TUNEL staining. All data are shown as mean ± SE of three independent experiments (# P<0.05, ## P<0.01, ### P<0.001, NAC, DIC pretreatment compared with TSA treatment alone, NQO1 siRNA pretreatment compared with control siRNA pretreatment, H596-NQO1 cells compared with H596-vector cells; ** P<0.01, *** P<0.001, TSA treatment compared with control cells).

TUNEL assays were performed to confirm that TSA induced cell death is due to the induction of NQO1 dependent apoptosis. As shown in Fig. 2A, TSA induced a concentration dependent apoptosis and the TUNEL positive cells reached over 80% upon 40 µM of TSA treatment in A549 cells, whereas the TUNEL positive cells detected from H596 cells upon TSA treatment is of no difference from control treatment. In addition, both DIC and NQO1 siRNA could significantly reverse TSA induced apoptosis in A549 cells (Fig. 2A, B). Likewise, the transfection of NQO1 to H596 cells significantly restored its susceptibility to TSA induced apoptosis, characterized with a 38.02% versus 7.28% TUNEL positive cells in H596-NQO1 cells and H596-vector cells (Fig. 2C), respectively. The results of TUNEL assays are in complete agreement with those from MTT based cytotoxicity studies, strongly suggesting that TSA induces an NQO1 dependent apoptotic cell death in NSCLC. Consistent with the MTT result, the TUNEL assay also showed that p53 inhibitor PFT-α and P53 siRNA had insignificant effect on TSA induced apoptosis, indicating a p53 independent apoptosis induced by TSA.

### TSA induces NQO1 dependent DNA damage

**Figure 3:**
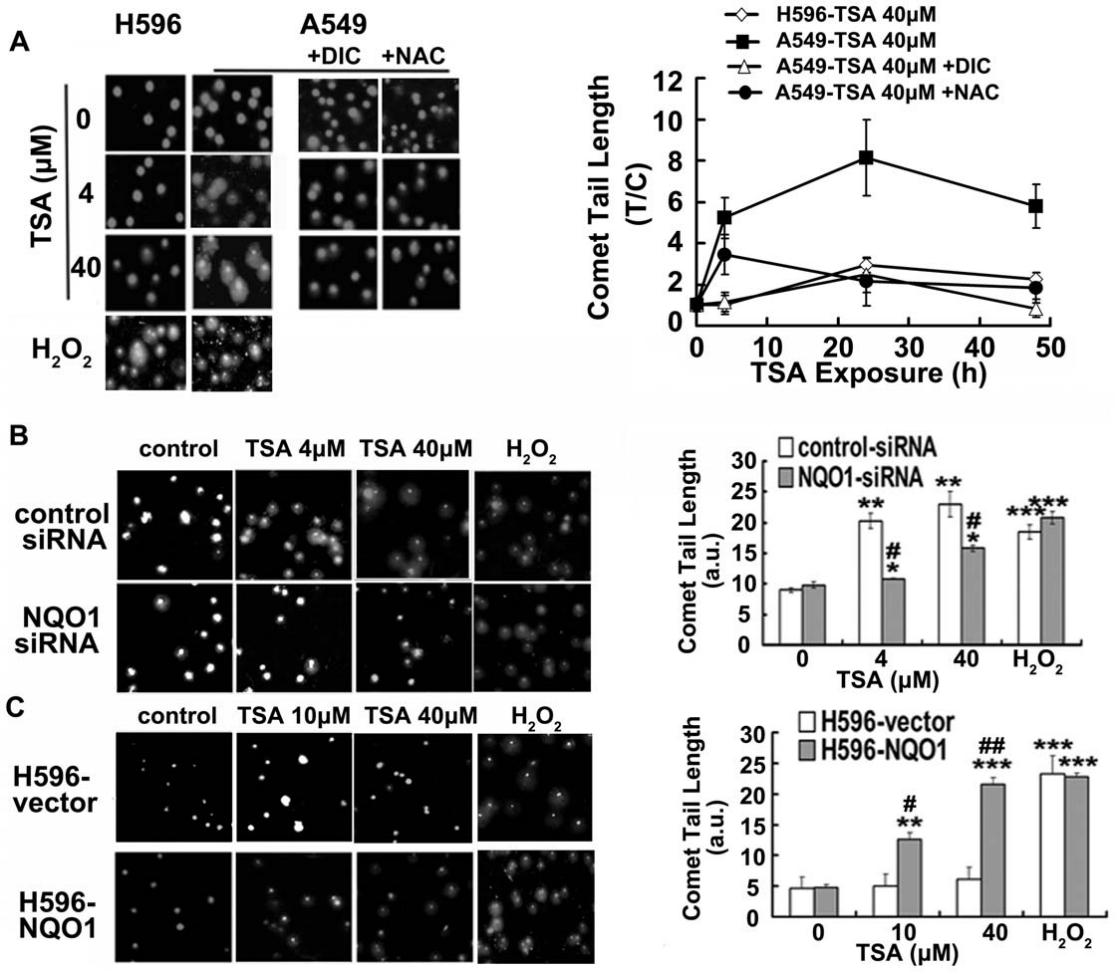
TSA induces NQO1-dependent and ROS mediated DNA damage. DNA lesions determined by alkaline comet assays. A, TSA induced DNA damage in A549 and H596 cells, as well as the effect of NAC or DIC pretreatment. B, effect of NQO1 silence in TSA induced DNA damage in A549 cells. C, DNA damage in H596-NQO1 and H596-vector cells. Left panel, the representative morphological appearance of cells with TSA treatment for 24 h; right panel, quantitative results expressed as fold change (T/C) of comet tail lengths or expressed as comet tail lengths (arbitrary unit). Data are shown as mean ± SE of at least three independent experiments. (# P<0.05, ## P<0.01 NQO1 silence vs. control siRNA or H596-NQO1 vs. H596-vector; * P<0.05, ** P<0.01, *** P<0.001, TSA or H_2_O_2_ treatment compared with control cells).

Because DNA damage is a pivotal initiator on inducing apoptotic cell death [34], we sought to determine whether TSA treatment induces NQO1-dependent DNA damage and breaks. As expected, TSA induced a dramatic and concentration dependent DNA damage in A549 and H596-NQO1 cells but minimal in NQO1 negative H596 and DIC or siRNA pretreated A549 cells (Fig. 3A, B and C). H_2_O_2_ (200 µM) treatment for 4 h induced almost identical DNA damage in all the cell lines. Time course data showed that the TSA induced NQO1-dependnet DNA damage reached the maximum after 24 h treatment but slightly decreased after 48 h, possibly because of excessive cell death (Fig. 3A). It was also noted that TSA at 40 µM induced quite similar amount of DNA damage in A549 cells and H596-NQO1 cells in spite of different NQO1 enzymatic activity between A549 cells and H596-NQO1 cells. We proposed that it is possibly that TSA at 40 µM may induced a maximum DNA damage for both A549 and H596-NQO1 cells and thus no difference was observed. However, A549 cells are indeed to be much more sensitive than H596-NQO1 cells to TSA induced DNA damage at lower dose challenge (Fig. 3B, C).

### ROS produced from NQO1 bioactivation is an important mediator on TSA induced apoptotic cell death

**Figure 4:**
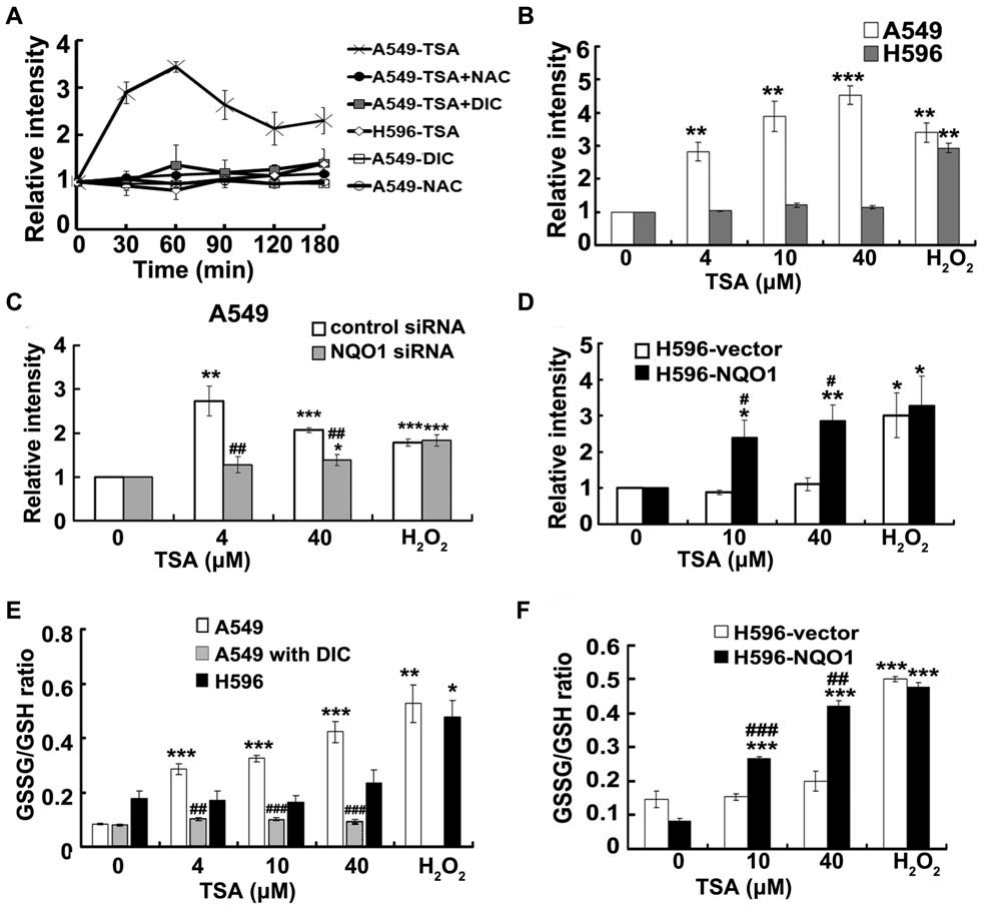
TSA induces NQO1 dependent generation of ROS in NSCLC cells. Intracellular ROS generation measured by DCF staining. A, for time course study, cells were treated with 10 µM of TSA with or without pretreatment by 10 µM of DIC or 5 mM of NAC; B, cells were exposed to indicated doses of TSA for 1 h; C, effect of NQO1 silence on TSA triggered ROS formation; cells were exposed to 4 µM or 40 µM of TSA for 1 h; D, H595-NQO1 cells and H596-vector cells were exposed to 10 µM or 40 µM of TSA for 1 h. H_2_O_2_ (400 µM) was used as a positive control. E and F, Glutathione cycling assays; E, NSCLC cells were exposed to indicated doses of TSA with or without DIC pretreatment for 24 h. F, H596-vector and H596-NQO1 cells were exposed to TSA for 24 h. Data are shown as relative changes to blank controls and expressed as mean ± SE of at least three independent experiments. (# P<0.05, ## P<0.01, ### P<0.001 NQO1 siRNA vs. control siRNA, H596-NQO1 vs. H596-vector, A549 with DIC vs. TSA alone; * P<0.05, ** P<0.01, *** P<0.001, TSA or H_2_O_2_ treatment vs. their respective control cells).

We have found that TSA was reduced by NQO1 to produce a highly unstable catechol intermediate which could be either conjugated with glucuronic acid in the presence of UDP-glucuronic acid transferases (UGTs) or auto-oxidizes back to TSA without them [31]. Since lack of UGTs was observed in NSCLC cells, it is reasonable to postulate that TSA can trigger a futile redox cycle producing excessive ROS in NQO1 positive cells. To support this consideration, DCF staining and glutathione cycling assay were applied to monitoring TSA induced ROS formation. Time course of DCF staining data showed that TSA (10 µM) induced excessive ROS formation within 30 min and reached saturation in 60 min in A549 cells but not in H596 and DIC pretreated A549 cells (Fig. 4A). NQO1 silence largely prevented TSA induced ROS formation in A549 cells (Fig. 4C). Consistently, the transfection of NQO1 to H596 cells restored the capability of TSA induced production of ROS from the futile cycle (Fig. 4D). Results obtained from the glutathione cycling assay (Fig. 4E, F) further supported that TSA induced ROS production in an NQO1 dependent manner. In contrast, H_2_O_2_ induced almost identical ROS production in all cases, independent of NQO1 expression.

In view that NQO1 triggered ROS production is a very early and continuous event, it is important to determine whether ROS produced from TSA quinone and catechol recycling is a key player in TSA induced apoptotic cell death. The pretreatment of A549 cells with 5 mM N-acetyl cysteine (NAC), a typical ROS scavenger, was sufficient to remove TSA induced intracellular ROS (Fig. 4A). NAC pretreatment could significantly prevent TSA induced cytotoxicity (Fig. 1C), apoptosis (Fig. 2A), and DNA damage (Fig. 3A). Moreover, in all test aspects, the reversing effects of NAC pretreatment are almost comparable with that achieved by DIC pretreatment. All the results indicated that ROS produced from NQO1 catalyzed recycling process is an important mediator on inducing apoptotic cell death of NSCLC cells.

### TSA caused NQO1 and ROS-mediated, p53 independent activation of mitochondrial pathway

**Figure 5:**
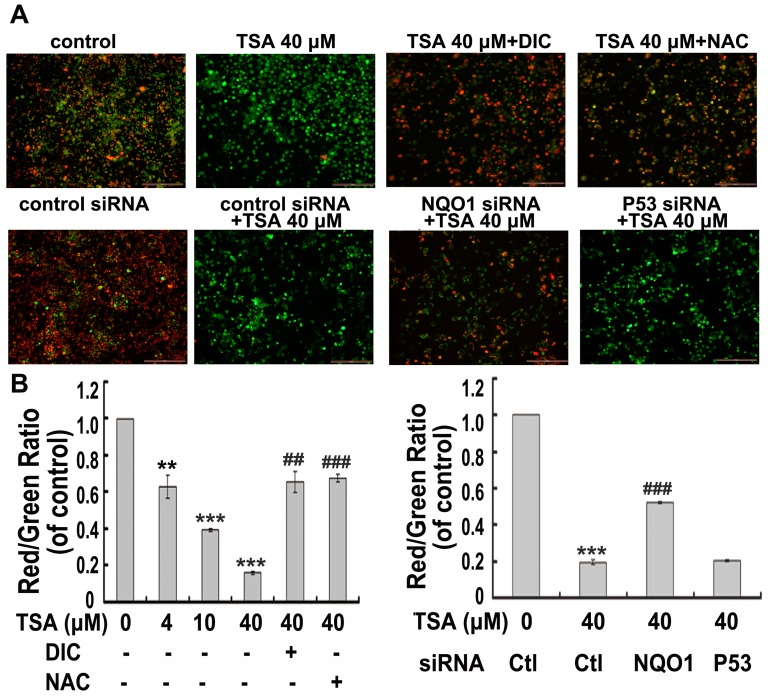
TSA activates an NQO1-initiated, ROS-mediated, and p53- independent mitochondrial membrane potential disruption (JC-1 assay). A549 cells were exposed to TSA of gradient concentrations (4, 10, and 40 µM) alone or pretreated with DIC, NAC, or subjected to NQO1 and p53 silence, prior to the treatment of 40 µM of TSA. Cells treated as indicated for 24 h were loaded with 3 µM of JC-1 for 15 min at 37°C, then softly washed with PBS for two times. In the cytosol, the monomeric form of this dye fluoresces green and within the mitochondrial matrix, highly concentrated JC-1 forms aggregates that fluoresce red. A, representative photos taken by Leica BMI3000 B microscope with a confocal microscope application. B, the fluorescence was read at 488 nm excitation and 530 nm emission for green, and at 540 nm excitation and 590 nm emission for red using a Synergy-H1 fluorimeter (Bio-Tek Instruments) and the changes in mitochondrial potential were calculated as the red/green ratio for each condition. (## P<0.01, ### P<0.001, DIC, NAC pretreatment compared with TSA treatment alone or NQO1 siRNA pretreatment compared with control siRNA pretreated cells; ** P<0.01, *** P<0.001, TSA treatment compared with control cells).

As shown in Fig. 5, TSA caused a dose-dependent disruption of MMP, which is consistent with a previous study by Chiu et al [35], in which TSA was found to induce a ROS mediated mitochondrial apoptotic cell death. Because we found herein that TSA induced apoptotic cell death was NQO1 dependent, we thus sought to determine the role of NQO1 in activating the mitochondrial apoptotic cell death pathway. NQO1 inhibitor DIC, NQO1 siRNA, as well as NAC pretreatment could significantly reverse TSA induced MMP disruption, indicating a NQO1 dependent and ROS-mediated mitochondrial disruption induced by TSA. P53 siRNA had no effect on TSA induced disruption of MMP, suggesting a p53-independent mechanism.

**Figure 6:**
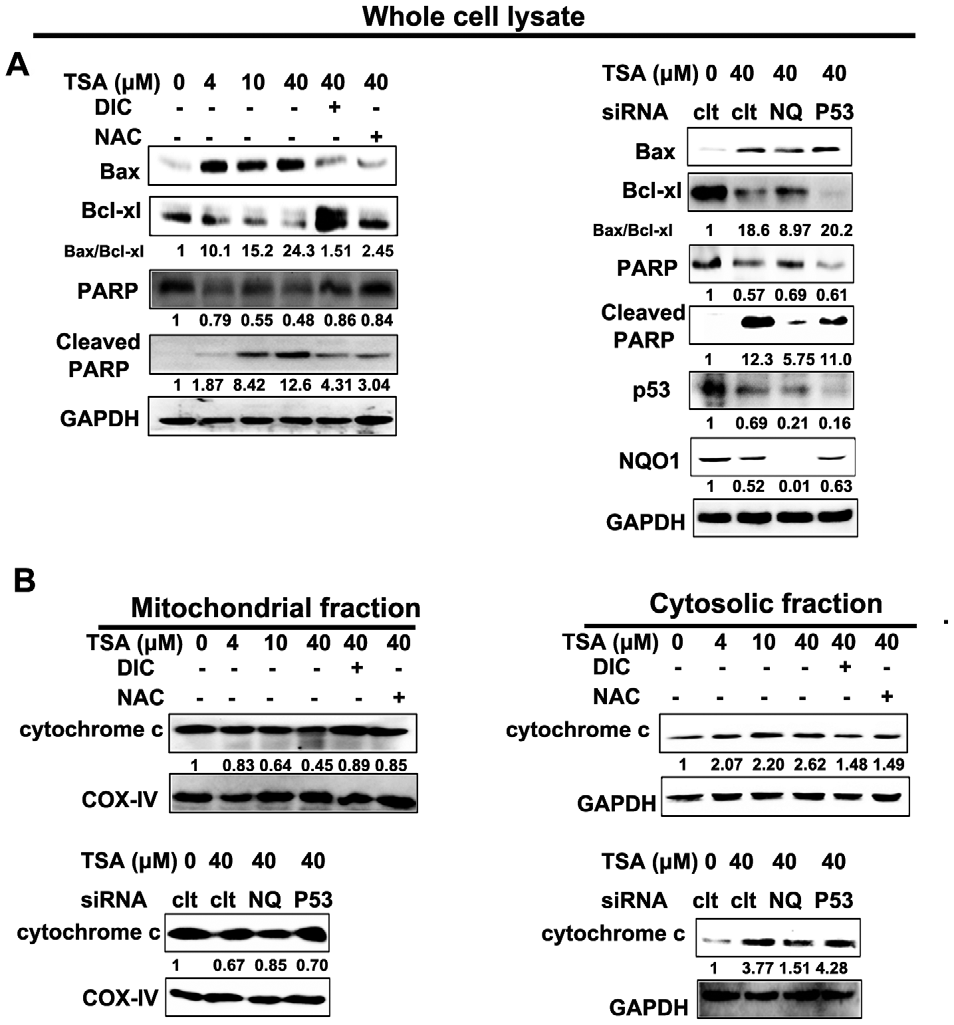
TSA activates an NQO1-initiated, ROS-mediated, and p53- independent mitochondrial apoptotic pathway. A549 cells were exposed to TSA of gradient concentrations (4, 10, and 40 µM) alone or pretreated with DIC, NAC, or subjected to NQO1 and p53 silence, prior to the treatment of 40 µM of TSA for 48 h. A, whole cell lysates were prepared after indicated treatment and western blot analysis was conducted using anti- Bax, -Bcl-xl, -PARP, -cleaved PARP, -NQO1, -p53, and -GAPDH antibodies. B, Cytosolic and mitochondrial proteins were prepared for western blot analysis using anti-cytochrome c antibody; fluctuations in protein loading between samples were monitored by GAPDH or COX-IV levels, respectively. The relative density value of each band is shown below the Western blot. The data are representative of a typical experiment that was conducted three times (mean values, P<0.05).

TSA treatment induced a time and concentration dependent upregulation of pro-apoptotic Bax and downregulation of pro-survival Bcl-xl in an NQO1 and ROS dependent but p53 independent manner (Fig. S1, Fig. 6A). This lead to a dramatic increased ratio of Bax/Bcl-xl, which is well known an important factor in governing cell apoptosis via the disruption of mitochondria [36]. Mitochondria disruption then lead to the release of cytochrome c from mitochondria, as evidenced from the cytosolic increase but mitochondrial decrease of cytochrome c (Fig. 6B).

**Figure 7:**
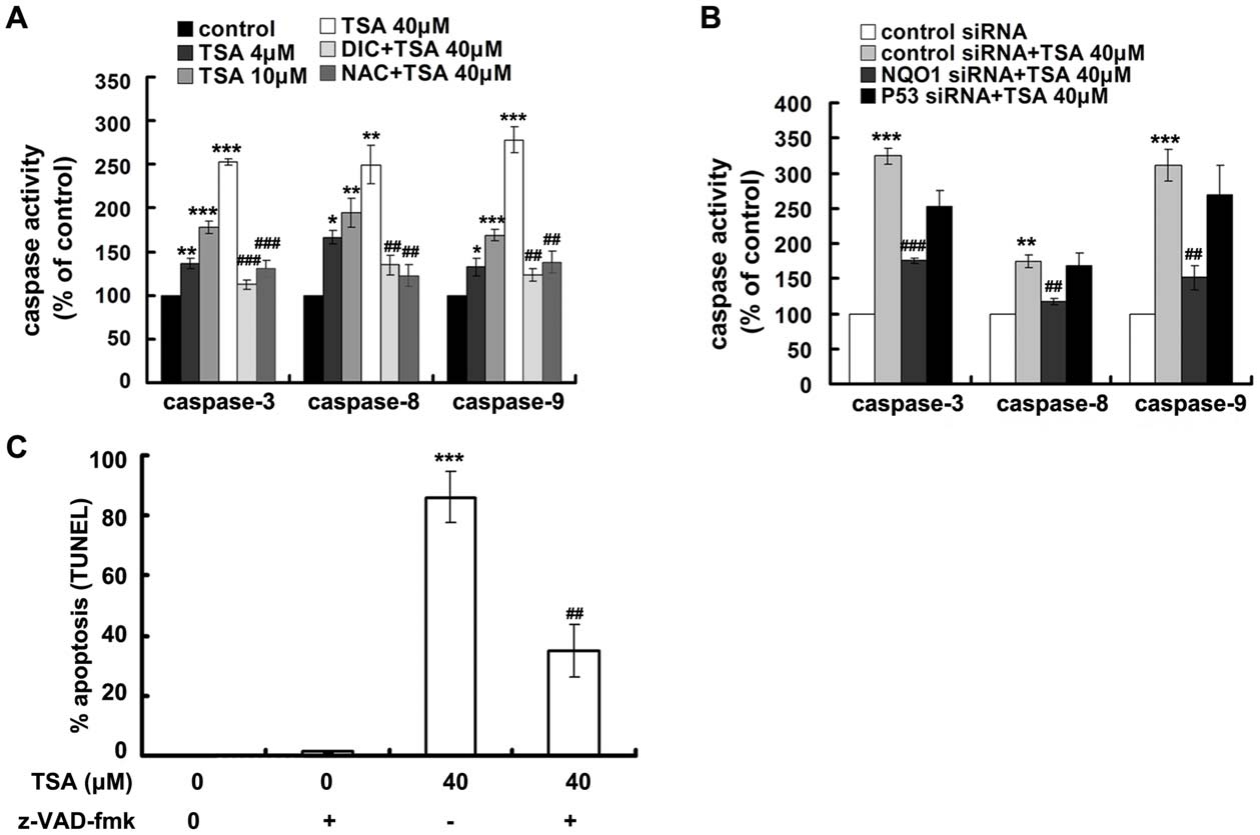
TSA caused caspase-dependent apoptosis. A and B, caspase activity test; A549 cells were exposed to TSA of gradient concentrations (4, 10, and 40 µM) alone or pretreated with DIC, NAC, or subjected to NQO1 and p53 silence, prior to the treatment of 40 µM of TSA for 48 h. C, TUNEL assay of TSA induced apoptosis with or without pretreatment of pan-caspase inhibitor z-VAD-fmk. Data are shown as mean ± SE of at least three independent experiments. (## P<0.01, ### P<0.001, DIC, NAC or z-VAD-fmk pretreatment compared with TSA treatment alone, NQO1 siRNA pretreatment compared with control siRNA pretreated cells; * P<0.05, ** P<0.01, *** P<0.001, TSA treatment compared with blank control cells).

Caspase activation was then analyzed by an activity assay using substrates of active cleaved caspase and a western blot analysis of PARP cleavage (Fig. 6A, and Fig. 7A, B). TSA dose dependently increased the activities of caspase-3, -9, and -8. The concentration dependent induction of PARP cleavage further supported caspase-3 activation. The pan-caspase inhibitor, z-VAD-fmk, largely attenuated TSA induced apoptosis (Fig. 7C). Altogether, these results suggest that TSA induced apoptotic cell death is caspase-mediated. Consistent with other aspects tested in our study, TSA induced collapse of MMP, cytochrome c release, and caspase activation were all NQO1 and ROS dependent but p53 independent.

### Antitumor effect of TSA in vivo is NQO1-dependent

**Figure 8:**
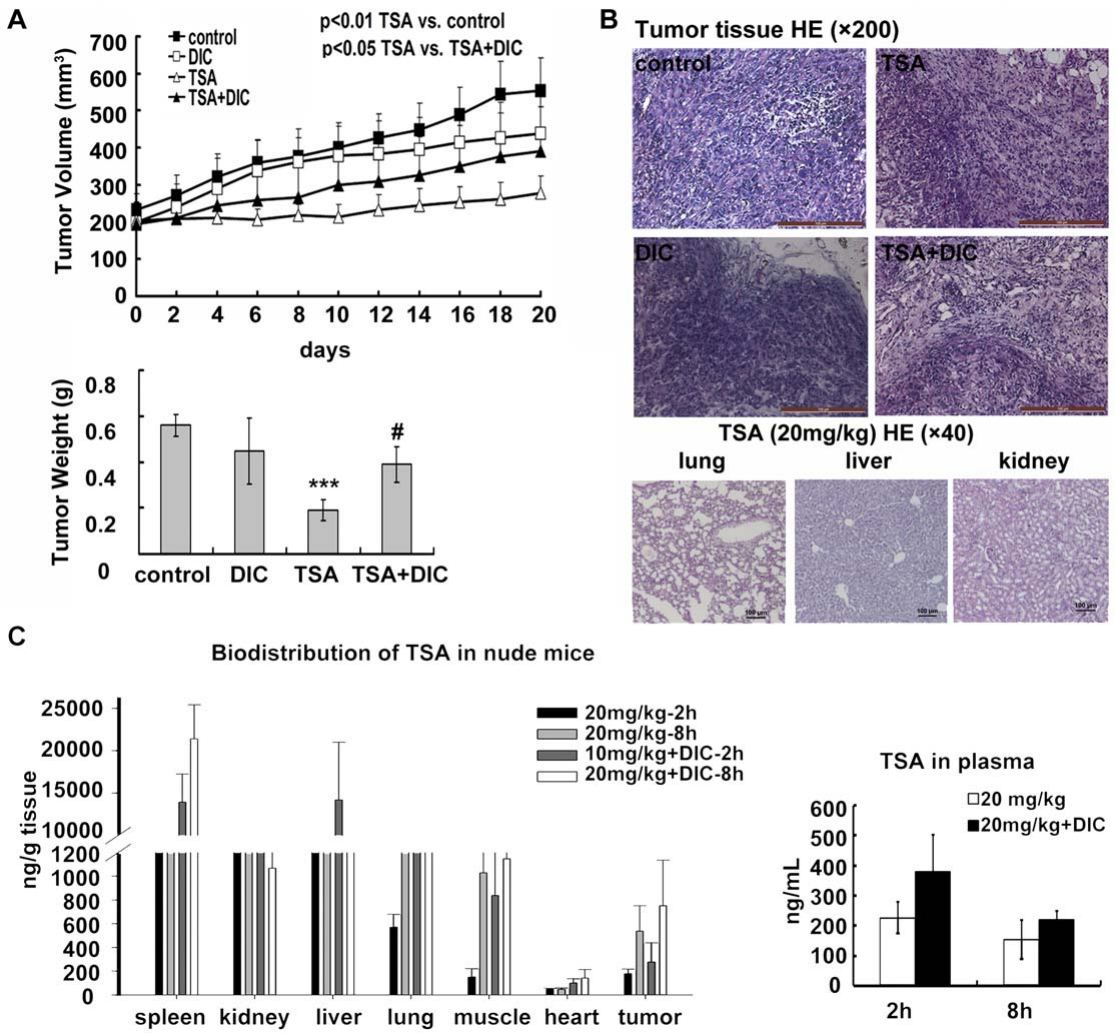
TSA retards the tumor growth NQO1 dependently in A549 tumor xenografted nude mice. A, upper, tumor volume measurement (P<0.01, TSA vs. control; P<0.05, TSA vs. TSA+DIC); lower, tumor weight (# P<0.05, TSA vs. TSA+DIC, *** P<0.001, TSA vs. control); data are shown as mean ± SE of six mice. B, histological examinations; tumor and tissue sections were analyzed by hematoxylin and eosin staining for histological features. C, biodistribution of TSA in A549 tumor loaded nude mice; data are shown as mean ± SE of 6 mice.

To confirm the role of NQO1, the antitumor efficacy of TSA was further investigated in A549 tumor xenografts by TSA treatment alone or in combination with DIC (Fig. 8A). TSA (20 mg/kg) treatment could significantly suppress the tumor growth; after 20 days treatment, the volume of tumors in TSA treated group was of 279±46 mm^3^ (P<0.01, compared with control group of 552±90 mm^3^). DIC treatment alone also slightly inhibited the tumor growth characterized with the tumor volume of 439±99 mm^3^; to the contrary, DIC pretreatment significantly antagonized the tumor suppression effect of TSA, suggesting that the antitumor effect of TSA in vivo was also NQO1 dependent. Histological examinations showed great tumor necrosis and apoptosis appeared in TSA treated group of mice, and of note, no toxicity in normal organ tissues was observed upon TSA treatment for 20 days (Fig. 8B).

To assess the tumor accumulation levels of TSA, tissue distribution study was performed in mice bearing A549 tumor xenografts (Fig. 8C). Consistent with our previous study of TSA distributions in rats [37], TSA that was prepared as a solid dispersion with PEG6000 preferentially distributed into the reticuloendothelial systems. Despite this limitation, TSA could still efficiently distribute into the tumor tissues; TSA showed higher accumulation levels and prolonged exposure times in tumors than that in plasma. DIC pretreatment, despite antagonizing the tumor suppression effect of TSA, significantly increased the exposure levels of TSA including that in tumor tissues, by a mechanism of inhibiting TSA metabolism via NQO1/UGTs [31]. Improving TSA delivery that can escape from reticuloendothelial system uptake would be expected to further increase its tumor accumulation levels and thus better anti-tumor efficacy.

## Discussion

Accumulating evidence suggests that NQO1 is a promising therapeutic target for various tumors, especially for NSCLC, in which NQO1 is overexpressed compared with normal lung tissue [14]. Nevertheless, there is currently no specific NQO1 target drugs used in clinic and thus its therapeutic value remains unexplored. Although some anti-tumor drugs such as mitomycin can be bioactivated by NQO1, all of them lack sufficient selectivity and specificity to NQO1 and their anti-tumor effects are better explained by other mechanisms [38], [39]. Evidence collected from the studies of Lap indicates that the NQO1 activated futile redox cycle can induce a unique cell death pathway, inspiring the new idea of developing NQO1 target anti-tumor drugs. This study contributes to identify a specific NQO1 substrate TSA, which induces a unique p53 independent activation of mitochondrial apoptotic pathway upon NQO1 bioactivation.

The hypothesis of NQO1 as the intracellular target of TSA was based on previous findings that NQO1 is the rate controlling enzyme in determining TSA metabolism [31], [32]. To verify it, we studied the role of NQO1 in determining the anti-cancer effects of TSA against NSCLC. In all tested aspects, TSA exhibited in an NQO1 dependent manner on inducing cytotoxicity, apoptosis, ROS production, and DNA damage; all of these events could be observed only in the NQO1 positive cell lines A549 and H596-NQO1. Consistently, the silence of NQO1 by specific inhibitor DIC and siRNA could largely abrogate all of these events in A549 cells. More importantly, the NQO1 dependent anti-cancer effect of TSA was further confirmed in the A549 tumor xenografts. TSA (20 mg/kg) treatment significantly retarded the tumor growth, whereas the co-administration of DIC could largely abrogate this effect. It was very interesting to note that DIC significantly increases the tumor accumulation of TSA while reduces its anti-tumor efficacy (Fig. 8). This can be explained by the fact that TSA was predominately metabolized by NQO1 and UGTs. However, NQO1 had little influence on the cellular uptake of TSA, as evidenced from the observation that the cellular accumulations of TSA in A549 and H596 cell lines was almost identical and the siRNA silence of NQO1 in A549 cells had little effect on TSA uptake (Fig. S2). Such a pharmacokinetic and pharmacodynamic paradox strongly indicates that the NQO1 mediated bioactivation is pivotal on determining TSA's anti-NSCLC efficacy.

The major characteristics of NQO1 catalyzed bioactivation is to produce a highly unstable catechol intermediate that auto-oxidizes to the parent quinone forming a futile redox cycle which renders cells to continuous ROS challenges. It has been recently reported that ROS produced from NQO1-mediated redox cycle played crucial role in Lap induced apoptosis [40]. It seems that ROS may also play an important role in TSA induced cell death, since NAC co-treatment could largely abolish TSA induced DNA damage, cell apoptosis, and cytotoxicity in NQO1 positive cells (Fig. 1, 2, and 3).

The p53 tumor suppressor is a pivotal regulating component that senses various intrinsic and extrinsic stresses and initiates apoptotic cell death [41]. Moreover, p53 protein degradation is negatively regulated by NQO1 via the inhibition of an ubiquitin-independent 20S proteosome mediated degradation pathway [42]. Therefore, it is important to determine the role of p53 on the apoptotic cell death induced by NQO1 substrates. All evidence collected from the present study indicated that TSA induced apoptotic cell death is p53 independent. First, the pretreatment with typical p53 inhibitor and the p53 silence by siRNA had negligible effect on TSA induced cytotoxicity, apoptosis, and all the tested events of mitochondrial apoptotic pathways in A549 cells (Fig. 1F, Fig. 2A, B, Fig. 5, Fig. 6, Fig. 7). Second, TSA could induce apparent and concentration dependent cytotoxicity and apoptosis in H596-NQO1 cells (Fig. 1E, 2C) in which the p53 is well known to be mutated [43]. Finally, TSA treatment in A549 cells induced a concentration dependent effect on decreasing p53 protein contents, and of interest, the NQO1 inhibitor DIC can synergize with TSA on decreasing p53 protein content in A549 cells but reverse the downregulation of NQO1 protein levels (Fig. S3). Although the detailed mechanisms underlying TSA's effect on down-regulating p53 protein levels warrant further research, the present results indicate that this effect may be caused by NQO1 regulated ubiquitin-independent p53 degradations, because TSA treatment also leaded to the down-regulation of NQO1 in parallel to the change of p53 protein levels (Fig. S3). NQO1 could stabilize p53 by preventing a ubiquitin-independent proteasomal degradation [15], [44]. Consistently, the NQO1 inhibitor like DIC could promote this degradation pathway and thus interfere with p53 initiated apoptotic pathway [45]. Our findings that TSA synergizing with DIC on reducing p53 protein levels provides reasonable explanation why NQO1 substrates induced apoptotic cell death is p53 independent. It may suggest that NQO1 substrates, like typical NQO1 inhibitors, can also promote the ubiquitin-independent proteasomal degradation; detailed mechanisms are currently under investigation in our laboratory.

**Figure 9:**
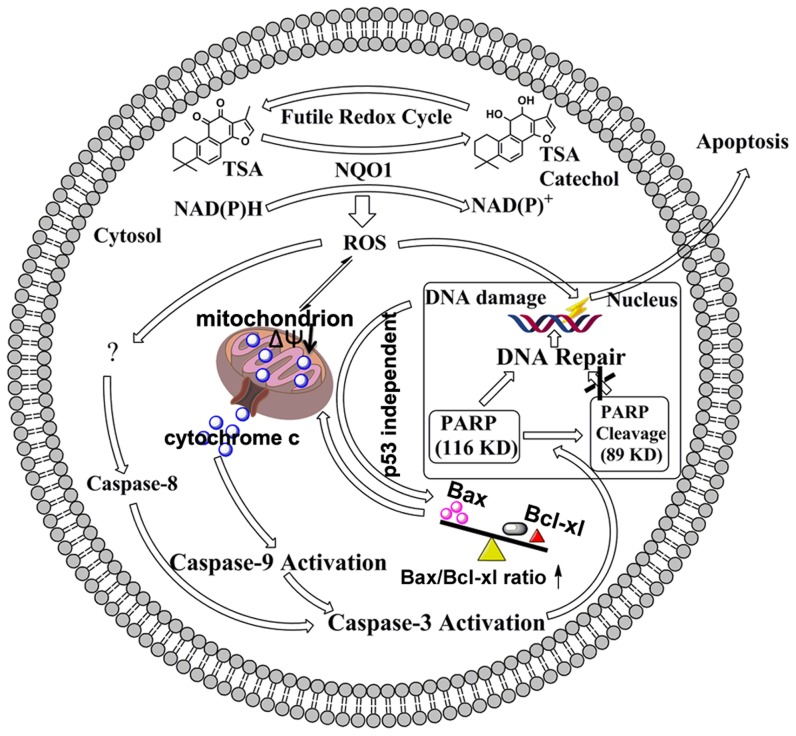
Proposed mechanisms for TSA-induced apoptotic cell death in NQO1-positive cells. TSA induces apoptotic cell death of NSCLC cells via a unique NQO1-initiated and ROS-mediated activation of a p53 independent but caspase dependent mitochondrial apoptotic pathway.

Since caspase serves as a central component of apoptosis machinery, we next sought to determine its role on TSA induced apoptotic cell death. Unlike the NQO1 substrate Lap which induced caspase independent apoptosis [46], TSA led to a concentration dependent activation of caspase-3, -8, and -9. In addition, pretreatment of the pan-caspase inhibitor z-VAD-fmk largely prevented TSA induced apoptosis that further demonstrated a caspase-dependent apoptosis upon TSA treatment (Fig. 7). Consistent with the results of apoptosis assays, the activation of caspases was also found also NQO1 and ROS dependent but p53 independent. Mitochondria mediated apoptosis is one crucial apoptotic mechanism involving activation of caspases. A previous study Chiu et al. demonstrated that TSA induced apoptosis of A549 cells was characterized with ROS production, DNA damage, and MMP loss [35], indicating that the ROS triggered mitochondrial pathway may play an important role on TSA induced cell death. For this consideration, we have made detailed studies herein to determine the role of NQO1 and p53 on initiating and programming the mitochondrial apoptotic cell death pathway. Our study showed that TSA treatment not only led to an NQO1 dependent and p53 independent loss of MMP and the subsequent release of cytochrome c from mitochondria (Fig. 5 and Fig. 6B), but also cause a p53 independent increased ratio of pro-apoptotic protein Bax to pro-survival protein Bcl-xl (Fig. 6A), which were considered to initiate apoptotic cell death [36]. These results in combination strongly suggest that the mitochondrial apoptotic pathway plays an important role on mediating TSA induced NQO1- and caspase-dependent but p53 independent apoptotic cell death in NSCLC cells. Although the detailed molecular mechanisms underlying TSA induced apoptosis warrant further research to explore, our results clearly indicate that some p53 backup systems can be activated by NQO1 triggered redox cycles and involving factors in the conditions where p53 is largely impaired due to NQO1 substrates induced instabilization. Taking together, it can be proposed that TSA is able to induce a p53 independent mitochondrial apoptotic pathway initiated by NQO1 and mediated by ROS (Fig. 9).

All aspects of in vitro studies indicate that TSA is a promising NQO1 target agent that may be developed to an effective therapeutic drug against NSCLC. To further validate the prospect of TSA development, the NQO1-dependent antitumor effect of TSA was investigated in vivo in A549 cells inoculated nude mice. TSA significantly inhibited the tumor growth (Fig. 8). DIC treatment alone could also partially inhibit the tumor growth, which is consistent with a previous report that DIC slowed the tumor growth and extended mice survival [47]. However, the DIC co-treatment could significantly reduce the antitumor activity of TSA. Although the antagonizing effect of DIC observed in vivo seems less strong than that observed in the in vitro studies, such a relatively weaker effect can support the NQO1 dependency of TSA in inhibiting the tumor growth, considering that DIC itself can partially retard the tumor growth and that DIC treatment increased the TSA accumulation levels in tumor tissues by inhibiting its metabolic eliminations (Fig. 8) [31]. Thus, both in vitro and in vivo data strongly support that TSA is a promising specific NQO1 target anti-tumor agent. In particular, TSA seems possess a wide therapeutic window because of its high NQO1 specificity and selectivity and its negligible toxicity in normal tissues (Fig. 8B). In the in vitro conditions, the NQO1 negative H596 cells are largely resistant to TSA induced toxicity even at a high concentration up to 80 µM and prolonged exposure times up to 72 h, which may represent a superior feature of TSA as compared with Lap (Fig. 1B) [18]. The in vivo histological examinations support that TSA has no toxic effects in normal tissues (Fig. 8). Our study showed that the NQO1 irrelevant cytotoxicity of TSA is much lower than that of Lap (Fig. 1B). TSA is a natural compound contained in Danshen, a Chinese medicine that have been widely used clinically for the therapy of various cardiovascular and cerebrovascular diseases; in addition, the TSA sodium sulphate derivative has been used in China as a cardiovascular drug for more than 20 years and no side effect of anemia has been found [48], [49], [50]. All lines of evidence hint to that TSA is unlikely to induce anemia that has been found for Lap; nevertheless, it is important in the future preclinical safety evaluation of TSA to monitor erythrocytes count. The major problem for TSA development as an effective anti-cancer drug may be of its poor oral bioavailability because of its poor water solubility and extensive intestinal first pass metabolism [31]. Nevertheless, some available pharmaceutical strategies such as TSA nanoemulsions [51] may improve its pharmacokinetic property and thus its druggability.

## Materials and Methods

### Ethics statement

All animal experiments were approved by the Animal Ethics Committee of China Pharmaceutical University. The use and care of experimental animals complied with all regulations in the Use of Laboratory Animals adopted and promulgated by the United States National Institutes of Health.

### Cell lines and Culture

Human NSCLC cell lines A549 (NQO1^+^) and NCI-H596 (NQO1^−^) were obtained from the American Type Culture Collection (ATCC, USA). Isogenically matched NQO1-expressing H596 cells were generated by infecting the log-phase H596 cells with lentiviral vectors expressing NQO1 or empty vector. Stable pooled populations Lenti6.3-NQO1-IRES-EGFP (H596-NQO1) or Lenti6.3-IRES-EGFP (H596-vector) were selected in 4.0 µg/mL of blasticidin S. HCL. Cells were grown in RPMI-1640 (Gibco, USA) medium with 10% fetal bovine serum (Hyclone, USA), 100 U/mL penicillin, and 100 µg/mL streptomycin (complete media) at 37°C in a humidified atmosphere with 5% CO_2_.

### Chemicals and Reagents

TSA was from the National Institute for the Control of Pharmaceutical and Biological Products (Beijing, China). Dicoumarol (DIC), N-acetyl cysteine (NAC), 2, 6-Dichlorophenolindophenol (DCPIP), β-lapachone (Lap), 2′,7′-Dichlorofluorescein diacetate (DCFH-DA), pifithrin-α (PFT-α), z-VAD-fmk, and Blasticidin. S. HCL were all from Sigma (St. Louis, MO, USA). Antibodies against NQO1 (epitomics, CA, USA), p53 (Millipore, MA, USA), GAPDH and β-actin (Boster Biology, Wuhan, China), Bcl-xl, Bax, PARP, cleaved PAPR, and cytochrome c (Cell Signaling Technology, MA, USA), and COXIV (beyotime, Jiangsu, China) were used.

### Measurement of NQO1 specific activity

NQO1 activity was measured as the rate of DIC-inhibitable DCPIP reduction in cell cytosolic samples as described previously [52]. The reaction was started by the addition of DCPIP, and the reduction of DCPIP was measured at room temperature for 2 min at 600 nm. The DIC-inhibitable part of DCPIP reduction was used to calculate NQO1 activity expressed as nmol DCPIP per mg protein per minute.

### Cytotoxicity assay

Exponentially growing cells were seeded at 8000 cells/well onto a 96-well plate (Costar) and kept overnight. The cells were incubated with TSA or Lap at indicated concentrations. After incubation for 72 h, 20 µL MTT (5 mg/mL) was added to each well and the plate was incubated at 37°C for another 4 h. MTT solution was removed and 150 µL DMSO was added per well; the absorbance at 570 nm was measured.

### Apoptosis assay

Apoptosis was quantified by using APO-BRDU™ Kit (BD Pharmingen, USA) according to the manufacturer's instruction. Samples were analyzed in a flow cytometer (BD FACSCalibur, USA) using an air-cooled argon laser at 488 nm. In view of the potential EGFP interference in NQO1 transfected H596 cells, the parallel TUNEL assay of H596-vector and H596-NQO1 cells were conducted using Click-iT TUNEL Alexa Fluor 594 Imaging Assay Kit (Molecular Probes, USA). Imaging was performed with Leica BMI3000 B microscope with a confocal microscope application.

### ROS assay

Intracellular ROS was analyzed from the conversion of nonfluorescent DCFH-DA to its fluorescent derivative. Fluorescence intensity was detected at 535 nm (with 488 nm excitation) in Synergy-H1 fluorimeter (Bio-Tek Instruments). Total glutathione and GSSG were measured from the reduction rate of 5,5′-dithio-bis (2-nitrobenzoic acid) (DTNB) using a commercial assay kits (Beyotime, Jiangsu, China). The absorbance changes at 412 nm were detected in an ultraviolet/visible spectrophotometer. GSSG to GSH ratio was calculated as an indicator of the intracellular oxidative stress.

### Alkaline Comet Assays

DNA lesions in single cells, including DNA single- and double-strand breaks, were assessed by alkaline comet assays. Slides were stained with ethidium bromide and visualized by using a fluorescence microscope (Nikon DS 5 M U1 CCD, Japan). Digital photomicrographs were taken and comet tails were quantified by analyzing at least 50 randomly selected comets per slide with the software (Resarch Biolab Co., ltd, Beijing, China).

### Mitochondrial Transmenbrane Potential measurement

The changes in mitochondrial membrane potential were monitored by 5,5′,6,6′-tetrachloro-1,1′,3,3′-tetraethyl-benzimidazolcarbocyanine iodide (JC-1) (KeyGen, Nanjing, China) staining. The green monomeric form of this dye in the cytosol and the red concentrated aggregates in the mitochondria were detected using a Synergy-H1 fluorimeter (Bio-Tek Instruments); the changes in mitochondrial potential were calculated as the red/green ratio for each condition.

### Caspase activity analysis

Caspase-3, caspase-8, and caspase-9 activities were measured by colorimetric assays kits (KeyGen, Nanjing, China). Cells post-treatment were lysed in chilled lysis buffer and were centrifuged at 15,000 g for 5 min; the supernatants were added to 50 µL of reaction buffer and 5 µL of respective caspase substrate. After incubation at 37°C for 4 h, absorbance was read at 405 nm.

### Transient NQO1 or P53 siRNA transfection

A549 cells were transfected with control siRNA or siRNA targeting NQO1 or P53 (invitrogen) using RNAiMAX transfection Reagent (Invitrogen) according to the manufacturer's instruction. Two days later, cells were harvested for western blot analysis to confirm the efficiency of siRNA knockdown.

### Western blot analysis

Cells were gently scraped with cell scrapers and subjected to total protein extraction. Protein concentration was then determined using the Bradford method. Equal amount of protein (50 µg) was loaded and separated by SDS-PAGE. After electrophoresis, proteins were transferred to a PVDF membrane (PALL, USA). Blots were blocked with 5% skim milk in TBST buffer and incubated overnight at 4°C with specific primary antibodies. After washing with TBST, the membrane was incubated with HRP-conjugated secondary antibody (KeyGen, Nanjing, China) for 1 h. The signal was visualized by enhanced chemiluminescence (ECL, Millipore). The protein expression levels were normalized with GAPDH or COX-IV to correct any experimental handing error.

### Subcellular fractionation

Cells were harvested and washed in ice-cold PBS and then resuspended in an isotonic buffer on ice for 20 min. Cells were then homogenized and centrifuged at 800× g for 10 min at 4°C. Supernatants were centrifuged at 14,000× g for 30 min at 4°C to obtain mitochondrial and cytosolic fractions. Mitochondrial fractions were lysed in 1% Chaps buffer for immunoblot analysis.

### In vivo anti-tumor efficacy

Log-phase A549 cells (5×10^6^) were injected s.c. into the flanks of athymic nude mice (aged 6–8 weeks). Tumor sizes were regularly measured using calipers, and volumes were calculated using the following formula: volume (mm^3^) = length×width×width/2. Animals were randomized into four groups (6 mice/group) when the average tumor size reached around 200 mm^3^ for the following treatments: group 1, vehicle; group 2, DIC (5 mg/kg); group 3, TSA (20 mg/kg); and group 4, DIC (5 mg/kg) 1 h prior to TSA (20 mg/kg) injection. All agents were administered every other day for 3 weeks through intraperitoneal administration. Tumor sizes were monitored and measured every other day. Differences in tumor volumes were analyzed statistically by comparing the slopes of the regression lines for plots of tumor volume vs. days of TSA treatment in mice receiving different treatments.

### Biodistribution study in A549 tumor inoculated nude mice

Mice randomized to six groups were sacrificed at 2 or 8 h after a single intraperitoneal administration of TSA at 20 mg/kg or a co-administration of 5 mg/kg of DIC with TSA (20 mg/kg), respectively. TSA levels in the plasma, major organ tissues, and tumors were determined by a LC-MS/MS method as previously reported [53].

### Statistical analysis

All data are presented as means ± SE of at least three samples and reproducibility was confirmed in three independent experiments. Statistical differences between two groups were evaluated using the Student's t-test; for multiple comparisons, one way analysis of variance followed by Student-Newman-Keuls Post Hoc test was applied. The difference was considered significant at *P<0.05, **P<0.01, or ***P<0.001.

## Supporting Information

Figure S1 **TSA induced time dependent increased ratio of Bax to Bcl-xl.** A549 cells were treated with 10 µM of TSA for indicated time, whole cell lysates were prepared and western blot were conducted using anti-Bax, anti-Bcl-xl and anti-GAPDH antibodies. The relative density value of each band is shown below the Western blot. The data are representative of a typical experiment that was conducted three times (mean values, P<0.05).
(TIF)

Figure S2 **Time and concentration dependent intracellular accumulations of TSA in NSCLC cells.** A, H596 versus A549 cell lines; B, control siRNA versus NQO1 siRNA transfected A549 cells. Confluent monolayer cells were preincubated in 1 mL of HBSS at 37°C for 30 min and then washed twice with HBSS before cellular uptake assay. Time dependent experiments were tested at TSA concentration of 10 µM and incubated for 15–210 min; concentration dependent accumulations were determined at TSA concentration range of 0–40 µM for 120 min. At the indicated time-points, cells were collected and lysed for determining the intracellular concentration of TSA by a previously developed and validated LC-MS/MS method. Protein concentration was measured by the Bradford assay and the uptake quantity was expressed as nmol/mg protein.
(TIF)

Figure S3 **p53 and NQO1 protein expression after exposure to TSA.** A549 cells were exposed to TSA of gradient concentrations (4, 10, and 40 µM) with or without pretreatment with 10 µM DIC for 30 min. whole cell lysates were prepared and western blot were conducted using anti-p53, anti-NQO1 and anti-GAPDH antibodies. The relative density value of each band is shown below the Western blot. The data are representative of a typical experiment that was conducted three times (mean values, P<0.05).
(TIF)
