# Supplementary figures and images for: An NQO1-Initiated and p53-Independent Apoptotic Pathway Determines the Anti-Tumor Effect of Tanshinone IIA against Non-Small Cell Lung Cancer
Source: PLoS One. 2012 Jul 27;7(7):e42138. doi: 10.1371/journal.pone.0042138 (PMC3407158; doi:10.1371/journal.pone.0042138)

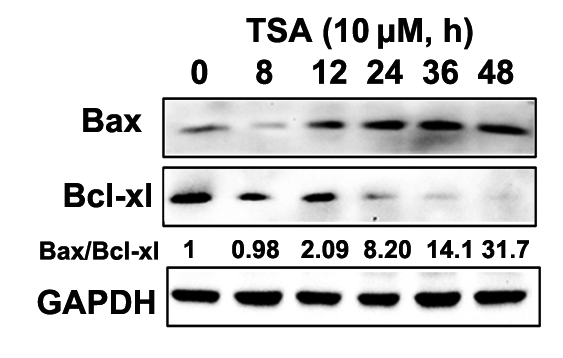

Supplement: Figure S1 — TSA induced time dependent increased ratio of Bax to Bcl-xl. A549 cells were treated with 10 µM of TSA for indicated time, whole cell lysates were prepared and western blot were conducted using anti-Bax, anti-Bcl-xl and anti-GAPDH antibodies. The relative density value of each band is shown below the Western blot. The data are representative of a typical experiment that was conducted three times (mean values, P<0.05). (TIF) [file pone.0042138.s001.tif]

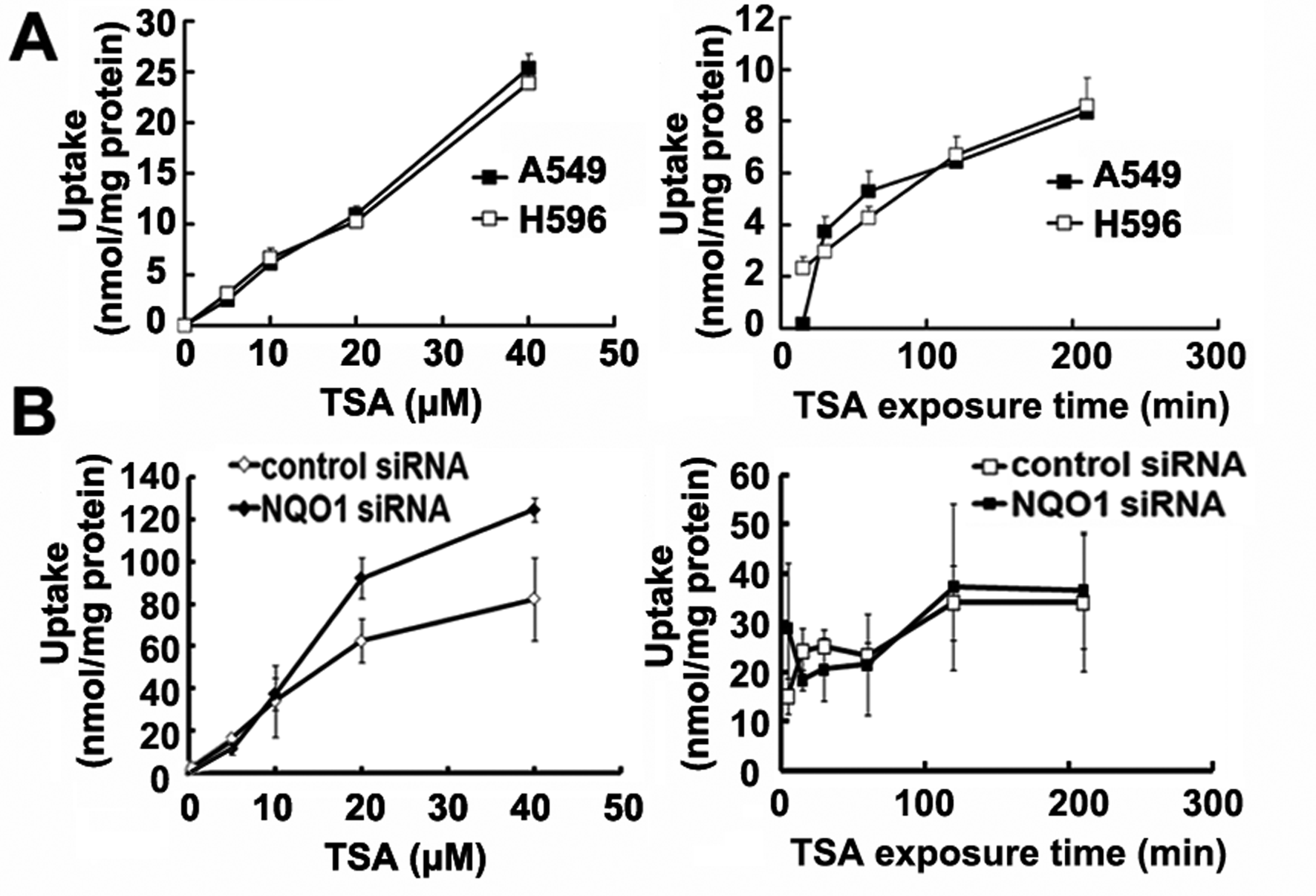

Supplement: Figure S2 — Time and concentration dependent intracellular accumulations of TSA in NSCLC cells. A, H596 versus A549 cell lines; B, control siRNA versus NQO1 siRNA transfected A549 cells. Confluent monolayer cells were preincubated in 1 mL of HBSS at 37°C for 30 min and then washed twice with HBSS before cellular uptake assay. Time dependent experiments were tested at TSA concentration of 10 µM and incubated for 15–210 min; concentration dependent accumulations were determined at TSA concentration range of 0–40 µM for 120 min. At the indicated time-points, cells were collected and lysed for determining the intracellular concentration of TSA by a previously developed and validated LC-MS/MS method. Protein concentration was measured by the Bradford assay and the uptake quantity was expressed as nmol/mg protein. (TIF) [file pone.0042138.s002.tif]

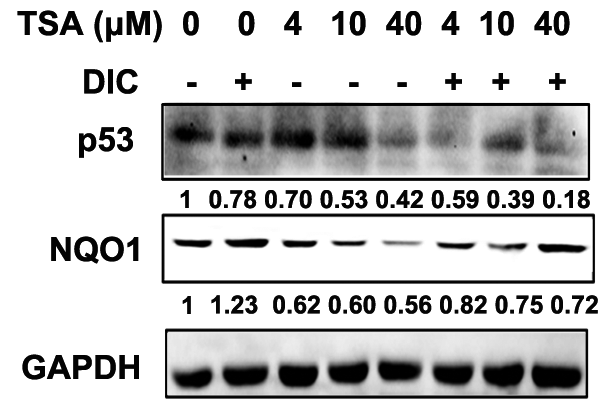

Supplement: Figure S3 — p53 and NQO1 protein expression after exposure to TSA. A549 cells were exposed to TSA of gradient concentrations (4, 10, and 40 µM) with or without pretreatment with 10 µM DIC for 30 min. whole cell lysates were prepared and western blot were conducted using anti-p53, anti-NQO1 and anti-GAPDH antibodies. The relative density value of each band is shown below the Western blot. The data are representative of a typical experiment that was conducted three times (mean values, P<0.05). (TIF) [file pone.0042138.s003.tif]
